# Effect of Tobacco Smoking Cessation on C-Reactive Protein Levels in A Cohort of Low-Dose Computed Tomography Screening Participants

**DOI:** 10.1038/s41598-018-29867-9

**Published:** 2018-08-27

**Authors:** Silvano Gallus, Alessandra Lugo, Paola Suatoni, Francesca Taverna, Elena Bertocchi, Roberto Boffi, Alfonso Marchiano, Daniele Morelli, Ugo Pastorino

**Affiliations:** 1 Department of Environmental Health Sciences, Istituto di Ricerche Farmacologiche Mario Negri IRCCS, Milan, Italy; 2 Thoracic Surgery Unit, Fondazione IRCCS Istituto Nazionale Tumori, Milan, Italy; 3 Immunohematology & Transfusion Medicine, Fondazione IRCCS Istituto Nazionale Tumori, Milan, Italy; 4 Tobacco Control Unit, Fondazione IRCCS Istituto Nazionale Tumori, Milan, Italy; 5 Department of Radiology, Fondazione IRCCS Istituto Nazionale Tumori, Milan, Italy; 6 Laboratory Medicine Unit, Fondazione IRCCS Istituto Nazionale Tumori, Milan, Italy

## Abstract

Smokers have higher levels of C-Reactive Protein (CRP) compared to never smokers. The role of smoking cessation on CRP is still under debate. Using data from two screening studies conducted in Italy in 2000–2010 on 3050 heavy smokers (including 777 ex-smokers), we estimated multivariate odds ratios (OR) for high CRP (i.e. ≥2 mg/L) according to smoking status. Moreover, in a longitudinal analysis based on 975 current smokers, with a second measurement of CRP after an average study period of 3.4 years, we estimated the changes in CRP according to smoking cessation. Prevalence of high CRP at baseline was 35.8% among ex-smokers and 41.1% among current smokers (significant OR for ex- vs. current smokers: 0.79). After four years since smoking cessation, CRP levels significantly decreased with increasing years of cessation (significant OR for ex-smokers since more than 8 years: 0.55). In the longitudinal analysis, no significant reduction in CRP was found for time since smoking cessation (ORs: 1.21, 1.04, and 0.91 for ex-smokers since 1 year, 2–3 years, and ≥4 years, respectively). In the largest prospective study available so far, we found that smoking cessation has a favourable effect on CRP, but this benefit is not evident in the short-term.

## Introduction

C-Reactive Protein (CRP) is synthesized by the liver in response to inflammation^1^. CRP test in human blood is one of the most common hematology tests to measure non-specific inflammation. In the absence of an acute phase of inflammation, the level of CRP is relatively stable. An elevated baseline inflammatory status, as measured by CRP level, has been shown to increase the risk of several chronic conditions, including cardiovascular diseases^2,3^, lung cancer^4^, and colorectal cancer^5^. Moreover, CRP levels are considered good long-term predictors of prognosis and relapse in patients with various chronic diseases, including colorectal cancer^5^, non-small-cell lung cancer (NSCLC)^6^, and respiratory^7,8^, gastrointestinal^5^, or cardiovascular diseases^2^. Using data from a prospective study of lung cancer screening participants from Italy, we already observed that individuals with elevated levels of CRP had a more than three-fold increased risk of overall mortality^9^. Thus, although reverse causation (i.e., the raised CRP levels result from (early) symptoms of a concomitant disease) should not be ruled out, this inflammatory biomarker seems able to select higher-risk individuals for morbidity and mortality.

A direct association between elevated levels of CRP, as well as other markers of inflammation, and cigarette smoking has been reported in several investigations^1,10–21^, most studies showing a dose-response relationship between CRP levels and smoking intensity and/or duration^14,17,18,21,22^.

Smoking cessation has been shown to induce immediate reduction in the levels of several inflammation markers^15,23^. With specific reference to CRP, various cross-sectional studies showed that ex-smokers have reduced CRP levels as compared to current smokers^11,13,16,19–22^, the reductions being, however, significant only after several years since cessation (i.e., 5 to 20 years)^11,13,19–22^. In particular, two studies on heavy smokers found no significant differences in CRP levels between current and short-term smoking cessation^23,24^. Only a couple of longitudinal studies are available on the issue, but considering a single CRP assessment at follow-up and a maximum follow-up period of one year^25,26^. A few additional studies with repeated CRP measurements included a relatively limited number of smokers and considered periods of a few weeks^15,23,24,27^ up to one year^28^ since smoking cessation.

To provide additional information on the issue, we analyzed data from two large studies, which provided CRP levels among samples of heavy smokers before and after smoking cessation, thus allowing us to compare variations in CRP levels among successful smoking quitters. To our knowledge, this study represents the largest prospective study on the issue, and the sole study able to evaluate the role of smoking cessation on CRP levels over more than one year.

## Materials and Methods

### Study population

In the present analysis, we considered data on heavy smokers aged 50 years or older who received low-dose computed tomography (LDCT) in the context of two Italian screening studies conducted from 2000 to 2010, whose details were previously described^29–31^. Briefly, the first investigation was a pilot study started in 2000, offering yearly LDCT for a minimum of 5 years to 1035 smokers aged ≥50 years, with a smoking history of at least 20 pack-years, and without a history of cancer over the last five years^29^. The second study, the Multicentre Italian Lung Detection (MILD), started in 2005 and included 4099 smokers (1190 were randomized to annual LDCT screening, 1186 to biennial LDCT screening and 1723 to the control group) with the same characteristics of the previous study^30^. 1723 participants who were randomized to the control group in the MILD trial were excluded from the current analysis. Similarly, subjects with missing CRP value at baseline (n = 331) were also excluded. Finally, 30 subjects participated in both the studies. Thus, we considered a total of 3050 ever smokers (2273 current and 777 ex-smokers) with available information on CRP at baseline.

The studies were approved by the Ethics Committee of the Istituto Nazionale Tumori (Milan, Italy) and performed in accordance with relevant guidelines and regulations. All the eligible patients signed an informed consent form before enrolment.

### Data collection at baseline

For each participant, information at baseline was collected on age, sex, smoking status (ex-smokers were participants who had quit smoking since at least one year), number of cigarettes smoked per day, age at starting smoking, age at stopping, lung function (i.e., the percent predicted forced expiratory volume in the first second of expiration, FEV_1_), and plasma level of CRP. Measured height (cm) and weight (kg) at baseline were available for a subgroup of participants (n = 2001). From height and weight we derived body mass index (BMI; kg/m^2^). From smoking intensity and smoking duration, we derived the average number of pack-years.

### Longitudinal analysis

The longitudinal analysis was based on a total of 975 current smokers at baseline with a second measurement of CRP and who did not have a diagnosis of lung cancer during follow-up. The second measurement of CRP was obtained after an average period of 3.4 years (SD 1.5 years; range 1.0–10.2 years). Smoking status and possible date at smoking cessation was assessed at each annual or biennial clinical visit during and after the study period by medical interview and a detailed self-administered questionnaire. Ex-smokers at follow-up were participants who had quit smoking since at least one year from the second CRP measurement.

### Statistical analysis

Cross-sectional analysis: median values and interquartile range (IQR) for CRP at baseline were provided. We analysed differences between CRP at baseline according to smoking status through parametric (i.e., t-test) and non-parametric tests (i.e., Wilcoxon test -on ranks- and test on medians). Since CRP did not follow a normal distribution, we showed the findings from the non-parametric analysis (test on medians), only. To show differences in CRP values according to smoking status, we used the beeswarm plot. We used the log scale, to take into account the skewness of CRP distribution.

We dichotomized CRP into low (<2 mg/L) and high (≥2 mg/L) level^9^ and estimated the multivariate odds ratios (OR) of high versus low CRP level using unconditional logistic regression model, after adjustment for sex, age, pack-years, and percentage of predicted FEV_1_. A second logistic regression model was considered after further allowance for BMI.

In order to take into account the variability of CRP, we also performed a multiple linear regression analysis. To ensure normality of CRP, we transformed its distribution using lambda = −0.1 (selected through a Box-Cox transformation). The transformed variable was used to estimate beta coefficient and p-values from a linear regression model adjusted for sex, age, average number of pack-years, and percentage predicted FEV_1_.

Longitudinal analysis: we estimated the ORs of increased versus decreased CRP levels during the follow-up according to smoking status at the second CRP measurement. ORs were derived through unconditional multiple logistic regression models after adjustment for sex, age, pack-years, and percentage predicted FEV_1_.

## Results

Table 1 shows the distribution of the study population, considered in the cross-sectional analysis, according to selected demographic, clinical, anthropometric and smoking characteristics collected at baseline.

**Table 1:** Percent distribution (%) of current and ex-smokers in the cross-sectional analysis, according to selected characteristics collected at baseline.

	N	Current smokers at baseline (%)	Ex-smokers at baseline (%)
Total (N)	3050	2273	777
Sex			
Men	2123	66.3	79.1
Women	927	33.7	20.9
p-value^a^		<0.001	
Age (years)			
<54	812	28.5	21.0
54–57	764	26.1	22.0
58–61	722	23.5	24.2
≥62	752	21.9	32.8
p-value^a^		<0.001	
FEV_1_ (%)			
<80	479	17.4	14.6
80–99	1122	40.4	35.4
≥100	1271	42.2	50.0
*Missing*	178		
p-value^a^		0.001	
BMI (kg/m^2^)			
<18.5	30	2.0	0.5
18.5–24.9	798	43.9	31.3
25.0–29.9	888	42.7	47.9
≥30	285	11.4	20.3
*Missing*	1049		
p-value^a^		<0.001	
Pack-years			
<40	1484	46.9	53.8
≥40	1566	53.1	46.2
p-value^a^		0.001	

^a^Differences between current and ex-smokers were tested using chi-square tests.

**Figure 1:**
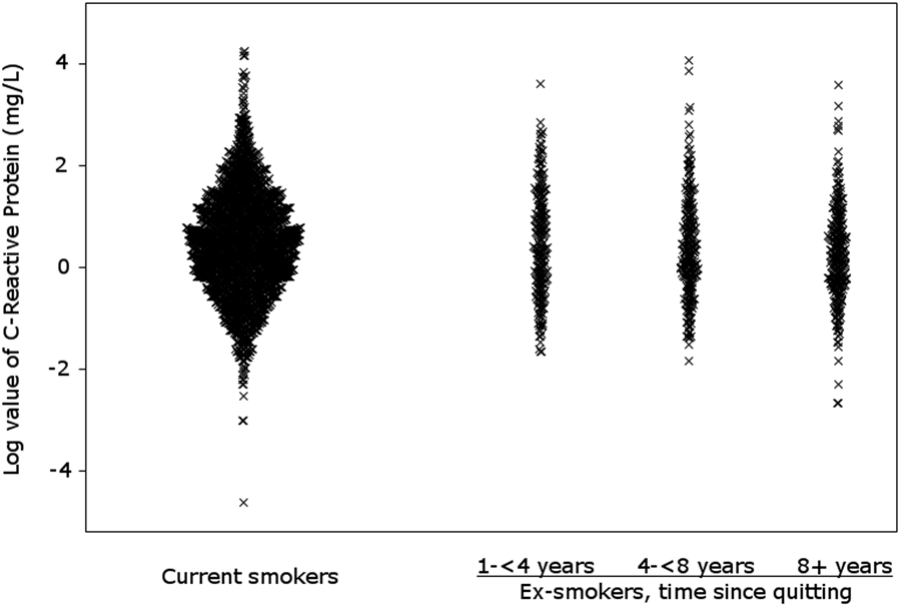
Beeswarm plot showing the distribution of C-Reactive Proteine (log scale) according to smoking status at baseline.

Mean CRP values at baseline was 2.84 mg/L among current smokers and 2.53 mg/L among ex-smokers. Median values were 1.61 mg/L (IQR: 0.82–3.18) and 1.35 mg/L (IQR: 0.73–2.75), respectively (p on medians <0.001). Median values of CRP decreased with increasing time since stopping, being 1.49 mg/L (IQR: 0.73–3.36) in ex-smokers since 1–3 years, 1.35 mg/L (IQR: 0.77–2.77) in 4–7 years and 1.22 mg/L (IQR: 0.73–2.24) in those having stopped since more than 8 years (Table 2). Among ex-smokers, the difference in median CRP was not significant for 4–7 vs. 1–3 years or ≥8 vs. 4–7 years, while the difference was statistically significant for ≥8 vs. 1–3 years since smoking cessation (p = 0.031). Figure 1 shows the beeswarm plot of CRP distribution by smoking status. Median values of CRP were significantly higher among men, subjects aged ≥65 years, with FEV_1_ < 80%, BMI ≥ 30 kg/m^2^, and pack-years smoked ≥40 **(**Supplementary Table 1).

**Table 2:** Odds ratios (OR) of C-Reactive Protein (CRP) ≥ 2 mg/L versus CRP <2 mg/L, and corresponding 95% confidence intervals (CI), according to smoking status and time since stopping smoking at baseline.

	N^a^	CRP, continuous variable	CRP, dichotomized variable		
		Median, mg/L	% CRP ≥ 2 mg/L	OR (95% CI)^b^ CRP ≥ 2 mg/L vs. <2 mg/L	OR (95% CI)^c^ CRP ≥ 2 mg/L vs. <2 mg/L
Smoking status					
Current smoker	2273	1.61	41.1	1^d^	1^d^
Ex-smoker	777	1.35	35.8	**0.79 (0.66–0.95)**	**0.59 (0.48–0.74)**
Time since stopping					
1–<4 years	242	1.49	42.6	1.09 (0.83–1.44)	0.86 (0.62–1.18)
4–<8 years	262	1.35	36.3	0.80 (0.61–1.06)	**0.55 (0.40–0.75)**
≥8 years	273	1.22	29.3	**0.55 (0.41–0.74)**	**0.41 (0.29–0.59)**
p for trend				<**0.001**	<**0.001**

IQR: interquartile range.

^a^Total number of ever smokers with available information on CRP at baseline (N = 3050).

^b^ORs were estimated using unconditional multiple logistic regression models after adjustment for sex, age, average number of pack-years, and percentage predicted FEV_1_. Estimates in bold are those statistically significant at the 0.05 level.

^c^ORs were estimated using unconditional multiple logistic regression models after adjustment for sex, age, average number of pack-years, percentage predicted FEV_1_ and body mass index. Estimates in bold are those statistically significant at the 0.05 level.

^d^Reference category.

Table 2 also shows that the overall prevalence of ever smokers with CRP ≥ 2 mg/L was 39.7%; ex-smokers were less likely to have CRP ≥ 2 mg/L compared to current smokers (OR was 0.79; 95% CI: 0.66–0.95). Compared to current smokers, OR for ex-smokers with time since stopping less than 4 years was 1.09 (95% CI: 0.83–1.44), for 4 to less than 8 years was 0.80 (95% CI: 0.61–1.06), and since more than 8 years was 0.55 (95% CI: 0.41–0.74). OR for ex-smokers since more than 5 years was 0.71 (95% CI: 0.57–0.89; data not shown in table). After further adjustment for BMI, the OR of ex smokers decreased to 0.59 (95% CI: 0.48–0.74). The ORs were 0.86 (95% CI: 0.62–1.18) for quitters since less than 4 years, 0.55 (95% CI: 0.40–0.75) for 4 to less than 8 years, and 0.41 (95% CI: 0.29–0.59) since more than 8 years. Similar results were found when we used, as an alternative cut-off point for high CRP, values ≥3 mg/L (Supplementary Table 2). The results from the dichotmized analyses were consistent with those found from multiple linear regressions: compared to current smokers, ex-smokers had a reduced CRP value (p < 0.001). However, CRP significantly decreased only after 8 years since stopping (p < 0.001). Once further adjusting for BMI, p-values became significant after 4 years since stopping smoking (p < 0.001; Supplementary Table 3). Table 3 shows the ORs for CRP ≥ 2 mg/L according to selected smoking characteristics, among ever, current, and ex-smokers. Among ever smokers, those with a higher smoking intensity (OR for ≥25 vs. <15 cigarettes per day was 1.65; 95% CI: 1.24–2.20; p for trend = 0.001), smoking duration (OR for ≥40 vs. <35 years of smoking was 1.42; 95% CI: 1.12–1.80; p for trend = 0.004), and number of pack-years (OR for ≥45 vs. <35 pack-years was 1.54; 95% CI: 1.27–1.86; p for trend < 0.001) were more likely to have CRP ≥ 2 mg/L. A similar pattern was observed among current smokers (corresponding ORs were 1.96, 95% CI: 1.41–2.72 for smoking intensity; 1.34, 95% CI: 1.00–1.78 for smoking duration, and 1.76, 95% CI: 1.48–2.22 for pack-years). Among ex-smokers, CRP was significantly higher in participants having smoked for more than 40 years (OR was 1.62; 95% CI: 1.03–2.57), while no significant relationship was observed between CRP and smoking intensity and number of pack-years.

**Table 3:** Odds ratios (OR) of C-Reactive Protein (CRP) ≥ 2 mg/L vs CRP <2 mg/L, and corresponding 95% confidence intervals (CI), according to selected smoking characteristics among ever, current, and ex-smokers at baseline.

Smoking characterisics	N^a^	% CRP ≥ 2 mg/L	OR (95% CI)^b^, CRP ≥ 2 mg/L vs <2 mg/L		
			Ever smokers	Current smokers	Ex-smokers
Smoking intensity (cigs/day)					
<15	306	30.7	1^c^	1^c^	1^c^
15–24	1749	38.6	**1.40 (1.07–1.83)**	**1.57 (1.15–2.13)**	0.92 (0.51–1.67)
≥25	995	44.4	**1.65 (1.24–2.20)**	**1.96 (1.41–2.72)**	0.91 (0.49–1.68)
p for trend			**0.001**	<**0.001**	0.807
Smoking duration (years)					
<35	877	32.8	1^c^	1^c^	1^c^
35–40	812	37.4	1.16 (0.93–1.44)	1.13 (0.87–1.47)	1.12 (0.74–1.67)
≥40	1361	45.5	**1.42 (1.12–1.80)**	**1.34 (1.00–1.78)**	**1.62 (1.03–2.57)**
p for trend			**0.004**	**0.047**	**0.047**
Pack years (number)					
<35	1013	31.2	1^c^	1^c^	1^c^
35–45	927	41.1	**1.36 (1.11–1.65)**	**1.53 (1.21–1.92)**	0.93 (0.62–1.39)
≥45	110	46.3	**1.54 (1.27–1.86)**	**1.76 (1.48–2.22)**	1.03 (0.71–1.50)
p for trend			<**0.001**	<**0.001**	0.858

^a^Total number of ever smokers with available information on CRP at baseline (N = 3050).

^b^ORs were estimated using unconditional multiple logistic regression models after adjustment for sex, age, percentage predicted FEV_1_, and, when applicable, smoking status. Estimates in bold are those statistically significant at the 0.05 level.

^c^Reference category.

Of the 975 current smokers at baseline with a second CRP measurement, 14.5% (n = 141) stopped smoking during the study period (Table 4). No significant association was found between smoking cessation and variation in CRP (OR of increased vs. decreased CRP for ex-smokers was 1.05; 95% CI: 0.73–1.51 compared to current smokers). No significant decrease in the OR was apparent with increasing of time since smoking cessation (ORs of increased vs. decreased CRP were 1.21, 1.04, and 0.91 for ex-smokers since 1 year, 2–3 years and ≥4 years, respectively). The results were similar when considering the ORs of subjects increasing or slightly decreasing CRP (i.e., by less than 20% of the value at baseline) vs. those considerably decreasing CRP (Supplementary Table 4).

**Table 4:** Odds ratios (OR) of increased versus decreased C-Reactive Protein (CRP), and corresponding 95% confidence intervals (CI), according to smoking status and time since stopping smoking in the longitudinal analysis.

	N^a^	% increased CRP	OR of increased vs. decreased CRP (95% CI)^b^
Smoking status at the end of the study period			
Current smoker	834	49.5	1^c^
Ex-smoker	141	50.4	1.05 (0.73–1.51)
Time since stoppimg			
1–<2 years	39	53.9	1.21 (0.63–2.33)
2–<4 years	67	50.8	1.04 (0.63–1.71)
≥4 years	35	45.7	0.91 (0.45–1.82)

^a^Total number of current smokers at baseline with available CRP measure at the end of the study period (N = 975).

^b^ORs refer to an increase in the value of CRP since the first measurement at the baseline to the second measurement at the follow-up, as compared to a decrease in CRP during this period. Subjects with the same value of CRP in both measurements were considered in the group of those who increased CRP. ORs for increased vs. decreased CRP at follow-up were estimated using unconditional multiple logistic regression models after adjustment for sex, age, average number of pack-years, and percentage predicted FEV_1_.

^c^Reference category.

## Discussion

In this study, we confirm that ex-smokers have significantly lower levels of CRP than current smokers^19^. CRP is significantly related to smoking intensity, duration and pack-years of smoking in ever and current smokers. Furthermore, we were able to elucidate the role of smoking cessation on CRP levels, confirming that a reduction in CRP is evident for former compared with current smokers. More importantly, we were also able to provide evidence, supported by longitudinal analyses, that no significant benefit in terms of CRP levels is evident at least during the first four years after cessation.

In ex-smokers, CRP appears to be significantly related to smoking duration, but not to intensity and pack-years. This supports the idea that CRP, as well as the risk of other diseases including lung cancer^32^, depends more strongly on smoking duration than on smoking intensity.

Most previous cross-sectional studies analyzing the issue found a relationship between CRP and smoking status, with current smokers having the highest levels of CRP, never smokers the lowest ones, and ex-smokers slowly reverting after smoking cessation to the levels of never smokers^11,13,16,19–22^.

Few other longitudinal studies are available on the issue. In one of these, based on a US cohort, authors compared CRP levels between 621 continuing smokers and 352 subjects who successfully stopped smoking. After one year of follow-up, CRP levels did not significantly differ among the two groups^25^. In another US cohort with a similar study design, based on 888 smokers attempting to stop, including 334 subjects who successfully stopped, after one year of follow-up, ex-smokers showed significant reductions in selected inflammatory markers, but not in CRP^26^. In another study, 46 female heavy smokers who successfully stopped smoking were followed for 6–7 weeks after smoking cessation. Several inflammatory bio-markers showed significant reductions; no statistically significant change was, however, observed for CRP^23^. In a randomized controlled study on 48 smokers, after only four weeks, suPAR values of ex-smokers fell to those of never smokers, but CRP levels did not significantly change^15^. Two other studies based on 154^24^ and 138 female smokers^27^, respectively, showed no significant variation in CRP levels between continuing smokers and successful quitters a few weeks after cessation. Finally, the same null result was found in a study based on 30 current smokers and 30 ex-smokers with available CRP measurements at baseline and after one year since smoking cessation^28^.

Various possible explanations can be given to explain the fact that smoking cessation appears to influence CRP in the long-term, but not in the short-term, as observed for several other biomarkers of inflammation^15,23^. For example, there is evidence that CRP levels are strongly related to various measures of adiposity and body fat, including waist circumference, BMI and weight gain^1,25,26,33^. Accordingly, BMI at baseline – available for a subgroup of our study population – showed a strong direct relationship with CRP levels (OR of CRP ≥ 2 mg/L was 5.26; 95% CI: 3.94–7.03, for obesity vs. normal weigh). Thus, the limited effect of smoking cessation on CRP may be explained by the fact that smoking merely affects cellular inflammation whereas CRP likely reflects metabolic, rather than cellular, inflammation^15,24^.

Moreover, there can be some residual confounding by unmeasured variables. In particular, it is possible that quitters more frequently develop smoking-related diseases or symptoms than continuing smokers^34^. Although we excluded from the longitudinal analysis subjects diagnosed with lung cancer between the two CRP measures, a residual confounding by concomitant diseases affecting inflammation should not be ruled out and may partly explain the unfavourable pattern for ex-smokers who quit smoking less than one year prior. More importantly, successful quitters gain weight rapidly after cessation^26,35^. Therefore, the possible inverse association between CRP and smoking cessation could have been masked by the stronger positive relaionship between CRP and weight gain^1,25^. Unfortunately, we had no information on BMI at follow-up and therefore we could not adjust our estimates for this variable in the longitudinal analysis. However, further adjustment for BMI in a subsample of study subjects in the cross-sectional analysis led to a substantial decrease in the ORs.

It is possible that CRP is a better indicator of overall mortality than of short-term morbidity. Indeed, the pattern of decline in CRP in relation to time since stopping smoking mirrors the risk curves observed for lung cancer incidence or total respiratory diseases and COPD mortality^36,37^. The CRP pattern unlikely mirrors the curves for coronary heart diseases, where significant reductions are already evident after 2 to 3 years since smoking cessation^13^.

Limitations of the present study include the impossibility to further adjust models of the longitudinal analysis for selected covariates, such as concomitant diseases or BMI assessed during the second CRP measurement; however, in the longitudinal analysis, further adjustment for BMI at baseline (as proxy of BMI at follow-up) did not modify the association between smoking status and change in CRP. We used data on self-reported smoking status without biochemical verification, thus the information is subject to a potential reporting bias. Nonetheless, previous reports have underlined the accuracy of self-reported measurements^38^. Moreover, we decided to dichotomize CRP using as the cut-off 2 mg/L. This may be subject to a degree of arbitrariness. However, the main results did not substantially differ changing the cut-off or considering CRP as a continuous variable. Finally, considering that the second CRP measurement occurred at a different time point, survival bias (i.e., people who stopped smoking for a long time are those who survived for such a period) could not be ruled out; however, the second CRP measurement was obtained within less than 5 years for 94% of subjects. Strengths include the longitudinal study design, the assessment of CRP measurements before and after cessation, and the uniquely large sample size – to our knowledge this is the largest longitudinal study in terms of sample size.

In conclusion, in the largest prospective study available so far, we confirm the favourable effect of smoking cessation on CRP levels, which increases with the length of time since stopping smoking. However, such benefit is not evident at least during the first four years following cessation. Our findings are compatible with the fact that CRP is a better indicator of (cancer) mortality rather than of short-term morbidity. However, the lack of any influence of smoking cessation on ex-smokers who quit less than four years prior may be partly or fully due to our impossibility to adjust longitudinal models for unmeasured covariates, including weight gain or BMI.

## Electronic supplementary material

Supplementary Tables

## References

[1] Emerging Risk FC (2010). C-reactive protein concentration and risk of coronary heart disease, stroke, and mortality: an individual participant meta-analysis. Lancet.

[2] Ridker PM (2016). A Test in Context: High-Sensitivity C-Reactive Protein. Journal of the American College of Cardiology.

[3] Danesh J (2004). C-reactive protein and other circulating markers of inflammation in the prediction of coronary heart disease. The New England journal of medicine.

[4] Chaturvedi AK (2010). C-reactive protein and risk of lung cancer. J Clin Oncol.

[5] Vermeire S, Van Assche G, Rutgeerts P (2005). The role of C-reactive protein as an inflammatory marker in gastrointestinal diseases. Nature clinical practice. Gastroenterology & hepatology.

[6] Leuzzi G (2016). Baseline C-reactive protein level predicts survival of early-stage lung cancer: evidence from a systematic review and meta-analysis. Tumori.

[7] Leuzzi, G. *et al*. C-reactive protein level predicts mortality in COPD: a systematic review and meta-analysis. *European respiratory review: an official journal of the European Respiratory Society* **26**, 10.1183/16000617.0070-2016 (2017). 10.1183/16000617.0070-2016 PMC9488765 28143876

[8] Chen YR (2017). C-reactive protein and N-terminal prohormone brain natriuretic peptide as biomarkers in acute exacerbations of COPD leading to hospitalizations. PLoS One.

[9] Pastorino, U. *et al*. Inflammatory status and lung function predict mortality in lung cancer screening participants. *European journal of cancer prevention: the official journal of the European Cancer Prevention Organisation (ECP)*, 10.1097/cej.0000000000000342 (2017). 10.1097/CEJ.0000000000000342 PMC6012047 28333763

[10] Kawada T (2015). Relationships between the smoking status and plasma fibrinogen, white blood cell count and serum C-reactive protein in Japanese workers. Diabetes Metab Syndr.

[11] Wannamethee SG (2005). Associations between cigarette smoking, pipe/cigar smoking, and smoking cessation, and haemostatic and inflammatory markers for cardiovascular disease. European heart journal.

[12] Ohsawa M (2005). CRP levels are elevated in smokers but unrelated to the number of cigarettes and are decreased by long-term smoking cessation in male smokers. Preventive medicine.

[13] Hastie CE, Haw S, Pell JP (2008). Impact of smoking cessation and lifetime exposure on C-reactive protein. Nicotine Tob Res.

[14] Bazzano LA, He J, Muntner P, Vupputuri S, Whelton PK (2003). Relationship between cigarette smoking and novel risk factors for cardiovascular disease in the United States. Ann Intern Med.

[15] Eugen-Olsen J, Ladelund S, Sorensen LT (2016). Plasma suPAR is lowered by smoking cessation: a randomized controlled study. European journal of clinical investigation.

[16] Shiels, M. S. *et al*. Cigarette smoking and variations in systemic immune and inflammation markers. *J Natl Cancer Inst* **106**, 10.1093/jnci/dju294 (2014). 10.1093/jnci/dju294 PMC4200029 25274579

[17] Conen D (2011). Smoking, smoking cessation, [corrected] and risk for symptomatic peripheral artery disease in women: a cohort study. Ann Intern Med.

[18] Lao XQ (2009). Smoking, smoking cessation and inflammatory markers in older Chinese men: The Guangzhou Biobank Cohort Study. Atherosclerosis.

[19] Tonstad S, Cowan JL (2009). C-reactive protein as a predictor of disease in smokers and former smokers: a review. Int J Clin Pract.

[20] Bakhru A, Erlinger TP (2005). Smoking cessation and cardiovascular disease risk factors: results from the Third National Health and Nutrition Examination Survey. PLoS Med.

[21] Lowe GD, Yarnell JW, Rumley A, Bainton D, Sweetnam PM (2001). C-reactive protein, fibrin D-dimer, and incident ischemic heart disease in the Speedwell study: are inflammation and fibrin turnover linked in pathogenesis?. Arteriosclerosis, thrombosis, and vascular biology.

[22] McEvoy JW (2015). Relationship of cigarette smoking with inflammation and subclinical vascular disease: the Multi-Ethnic Study of Atherosclerosis. Arteriosclerosis, thrombosis, and vascular biology.

[23] Reichert V (2009). A pilot study to examine the effects of smoking cessation on serum markers of inflammation in women at risk for cardiovascular disease. Chest.

[24] Aldaham S, Foote JA, Chow HH, Hakim IA (2015). Smoking Status Effect on Inflammatory Markers in a Randomized Trial of Current and Former Heavy Smokers. Int J Inflam.

[25] Asthana A (2010). Effects of smoking intensity and cessation on inflammatory markers in a large cohort of active smokers. American heart journal.

[26] King CC (2017). Longitudinal Impact of Smoking and Smoking Cessation on Inflammatory Markers of Cardiovascular Disease Risk. Arteriosclerosis, thrombosis, and vascular biology.

[27] Hammett CJ (2007). Variation in blood levels of inflammatory markers related and unrelated to smoking cessation in women. Prev Cardiol.

[28] Crook MA (2000). Circulating concentrations of C-reactive protein and total sialic acid in tobacco smokers remain unchanged following one year of validated smoking cessation. European journal of clinical investigation.

[29] Pastorino U (2003). Early lung-cancer detection with spiral CT and positron emission tomography in heavy smokers: 2-year results. Lancet.

[30] Pastorino U (2012). Annual or biennial CT screening versus observation in heavy smokers: 5-year results of the MILD trial. European journal of cancer prevention: the official journal of the European Cancer Prevention Organisation (ECP).

[31] Pastorino U (2016). Stopping Smoking Reduces Mortality in Low-Dose Computed Tomography Screening Participants. J Thorac Oncol.

[32] Peto, R. Influence of dose and duration of smoking on lung cancer rates. *IARC Sci Publ*, 23–33 (1986). 3623669

[33] Festa A (2000). Chronic subclinical inflammation as part of the insulin resistance syndrome: the Insulin Resistance Atherosclerosis Study (IRAS). Circulation.

[34] Gallus S (2013). Why do smokers quit?. European journal of cancer prevention: the official journal of the European Cancer Prevention Organisation (ECP).

[35] Gallus S (2013). Overweight and obesity prevalence and determinants in Italy: an update to 2010. Eur J Nutr.

[36] Li Y, Yamagishi K, Yatsuya H, Tamakoshi A, Iso H (2012). Smoking cessation and COPD mortality among Japanese men and women: the JACC study. Preventive medicine.

[37] Kenfield SA, Stampfer MJ, Rosner BA, Colditz GA (2008). Smoking and smoking cessation in relation to mortality in women. JAMA.

[38] Hatziandreu EJ (1989). The reliability of self-reported cigarette consumption in the United States. Am J Public Health.

