# Supplementary material for: Effect of Tobacco Smoking Cessation on C-Reactive Protein Levels in A Cohort of Low-Dose Computed Tomography Screening Participants
Source: Sci Rep. 2018 Aug 27;8:12908. doi: 10.1038/s41598-018-29867-9 (PMC6110802; doi:10.1038/s41598-018-29867-9)
Supplement: Supplementary file 1 — Supplementary Tables [file 41598_2018_29867_MOESM1_ESM.docx]

**EFFECT OF TOBACCO SMOKING CESSATION ON C-REACTIVE PROTEIN LEVELS IN A COHORT OF LOW-DOSE COMPUTED TOMOGRAPHY SCREENING PARTICIPANTS**

Silvano GALLUS ScD, Alessandra LUGO PhD, Paola SUATONI MD,

Francesca TAVERNA PhD, Elena BERTOCCHI MSc, Roberto BOFFI MD,

Alfonso MARCHIANO MD, Daniele MORELLI ScD, Ugo PASTORINO MD

**Supplementary Table 1**. Baseline median values of C-Reactive Protein (CRP) according to selected characteristics.

|  |  | N | Median CRP (IQR), mg/L | p-value^a^ |
| --- | --- | --- | --- | --- |
| Total (N) |  | 3050 | 1.53 (0.80-3.07) | - |
| Sex |  |  |  |  |
| Men |  | 2123 | 1.63 (0.85-3.22) | <0.001 |
| Women |  | 927 | 1.24 (0.66-2.66) |  |
| Age (years) |  |  |  |  |
| <54 |  | 812 | 1.26 (0.64-2.57) | <0.001 |
| 54-57 |  | 764 | 1.51 (0.79-2.99) |  |
| 58-61 |  | 722 | 1.63 (0.86-3.31) |  |
| ≥62 |  | 752 | 1.73 (0.91-3.47) |  |
| FEV1 (%)^b^ |  |  |  |  |
| <80 |  | 479 | 2.05 (1.11-4.29) | <0.001 |
| 80-99 |  | 1122 | 1.62 (0.88-3.30) |  |
| ≥100 |  | 1271 | 1.24 (0.65-2.50) |  |
| BMI (kg/m^2^)^b^ |  |  |  |  |
| <18.5 |  | 30 | 0.74 (0.30-1.88) | <0.001 |
| 18.5-24.9 |  | 798 | 1.04 (0.58-2.09) |  |
| 25.0-29.9 |  | 888 | 1.72 (0.95-3.20) |  |
| ≥30 |  | 285 | 2.70 (1.53-4.56) |  |
| Pack-years |  |  |  |  |
| <40 |  | 1484 | 1.30 (0.68-2.59) | <0.001 |
| ≥40 |  | 1566 | 1.79 (0.91-3.51) |  |

IQR: interquartile range.

^a^ Non-parametric test on medians.

^b^ The sum does not add up to the total because of some missing values.

**Supplementary Table 2**. Odds ratios (OR) of C-Reactive Protein (CRP) ≥3 mg/L versus CRP <3 mg/L, and corresponding 95% confidence intervals (CI), according to smoking status and time since stopping smoking at baseline.

|  | N^a^ | % CRP ≥3 mg/L | OR (95% CI)^b^  CRP ≥3 mg/L vs. <3 mg/L | OR (95% CI)^c^  CRP ≥3 mg/L vs. <3 mg/L |
| --- | --- | --- | --- | --- |
| Smoking status |  |  |  |  |
| Current smoker | 2273 | 27.1 | 1^d^ | 1^d^ |
| Ex-smoker | 777 | 22.4 | **0.76 (0.62-0.93)** | **0.63 (0.50-0.80)** |
| Time since stopping |  |  |  |  |
| 1- <4 years | 242 | 27.7 | 1.04 (0.76-1.41) | 0.89 (0.63-1.26) |
| 4- <8 years | 262 | 22.9 | 0.78 (0.57-1.06) | **0.57 (0.40-0.81)** |
| ≥8 years | 273 | 17.2 | **0.52 (0.36-0.74)** | **0.45 (0.29-0.68)** |
| p for trend |  |  | **<0.001** | **<0.001** |

^a^ Total number of ever smokers with available information on CRP at baseline (N=3050).

^b^ ORs were estimated using unconditional multiple logistic regression models after adjustment for sex, age, average number of pack-years, and percentage predicted FEV_1_. Estimates in bold are those statistically significant at the 0.05 level.

^b^ ORs were estimated using unconditional multiple logistic regression models after adjustment for sex, age, average number of pack-years, percentage predicted FEV_1_ and body mass index. Estimates in bold are those statistically significant at the 0.05 level.

^d^ Reference category.

**Supplementary Table 3**. Multiple linear regression model for C-Reactive Protein (CRP; continuous variable) according to smoking status and time since stopping smoking at baseline.

|  | Median CRP (IQR), mg/L | beta^a^ | p- value^a^ | beta^b^ | p- value^b^ |
| --- | --- | --- | --- | --- | --- |
| Smoking status |  |  |  |  |  |
| Current smoker | 1.61 (0.82-3.18) | ref. |  | ref. |  |
| Ex-smoker | 1.35 (0.73-2.75) | **-0.109** | **0.007** | **-0.251** | **<0.001** |
| Time since stopping |  |  |  |  |  |
| 1- <4 years | 1.49 (0.73-3.36) | 0.012 | 0.854 | -0.123 | 0.060 |
| 4- <8 years | 1.35 (0.77-2.77) | -0.096 | 0.126 | **-0.280** | **<0.001** |
| ≥8 years | 1.22 (0.73-2.24) | **-0.244** | **<0.001** | **-0.360** | **<0.001** |
| p for trend |  | **<0.001** | | **<0.001** | |

^a^ coefficients were estimated using multiple linear regression models after adjustment for sex, age, average number of pack-years, and percentage predicted FEV_1_. Since dependent variable was not normal, we transformed it using lambda=-0.1 selected through a Box-Cox transformation. Estimates in bold are those statistically significant at the 0.05 level.

^b^ coefficients were estimated using multiple linear regression models after adjustment for sex, age, average number of pack-years, percentage predicted FEV_1_ and body mass index. Since dependent variable was not normal, we transformed it using lambda=-0.1 selected through a Box-Cox transformation. Estimates in bold are those statistically significant at the 0.05 level.

**Supplementary Table 4**. Odds ratios (OR) of subjects increasing or slightly decreasing versus those considerably decreasing^a^ C-Reactive Protein (CRP), and corresponding 95% confidence intervals (CI), according to smoking status and time since stopping smoking in the longitudinal analysis.

|  | N ^b^ | % subjects who increased or slight decreased CRP | OR of subjects increasing or slightly decreasing versus those considerably decreasing CRP (95% CI)^c^ |
| --- | --- | --- | --- |
| Smoking status at the end of the study period |  |  |  |
| Current smoker | 834 | 61.9 | 1^c^ |
| Ex-smoker | 141 | 59.6 | 0.93 (0.64-1.35) |
| Time since stoppimg |  |  |  |
| 1-<2 years | 39 | 64.1 | 1.13 (0.58-2.23) |
| 2-<4 years | 67 | 62.7 | 1.01 (0.60-1.70) |
| ≥4 years | 35 | 48.6 | 0.65 (0.32-1.30) |

^a^ Subjects whose change in CRP between follow-up and baseline increased or decreased by less than 20% or increased.

^b^ Total number of current smokers at baseline with available CRP measure at the end of the study period (N=975).

^c^ ORs refer to an increase (or slight decrease) in the value of CRP since the first measurement at the baseline to the second measurement at the follow-up, as compared to a considerable decrease in CRP during this period. ORs were estimated using unconditional multiple logistic regression models after adjustment for sex, age, average number of pack-years, and percentage predicted FEV1.

^c^ Reference category.
